# Supplementary material for: High spatiotemporal resolution optoacoustic sensing with photothermally induced acoustic vibrations in optical fibres
Source: Nat Commun. 2021 Jul 6;12:4139. doi: 10.1038/s41467-021-24398-w (PMC8260642; doi:10.1038/s41467-021-24398-w)
Supplement: Supplementary file 1 — Supplementary Information [file 41467_2021_24398_MOESM1_ESM.pdf]

Supplementary Information:

High spatiotemporal resolution optoacoustic sensing with photothermally induced acoustic vibrations in optical fibres

Liang et al.

Supplementary Figure 1 Effect of the acoustic source size on the spatial resolution capability.

Supplementary Figure 2 Dispersion curves of the torsional-radial TR_{2n} modes.

Supplementary Figure 3 Calculated dispersion diagram and spatial mode profile of the TR_{2n} PTAVs in water.

Supplementary Figure 4 Acoustic spectra as a function of surrounding impedance.

Supplementary Figure 5 Temporal waveforms and frequency spectra of the excited PTAVs in water and saturated NaCl solution.

Supplementary Figure 6 Calculated real and imaginary parts in the denominator of Eq. S-21, analogous to frequency detuning and damping terms, respectively.

Supplementary Figure 7 Experimental setup for PTAV detection using the built-in laser.

Supplementary Figure 8 Demonstration of the sensitivity/sensing length trade-off of the PTAV detector.

Supplementary Figure 9 Mode analysis of the side-polished fibre.

Supplementary Table 1 Definition of terms for the calculation of fibre vibrational modes.

Supplementary Note 1 Optical-to-acoustic efficiencies of electrostriction and photothermal excitation.

Supplementary Note 2 Modelling of the optoacoustic sensing: a matrix method.

Supplementary Note 3 Vibrational modes of a cylindrical optical fibre.

Supplementary Note 4 Calculation of the sensitivity to a thickness change of the coating based on perturbation theory.

Supplementary Note 5 Optoacoustic sensing in liquids.

Supplementary Note 6 PTAV detection by using the built-in fibre laser.

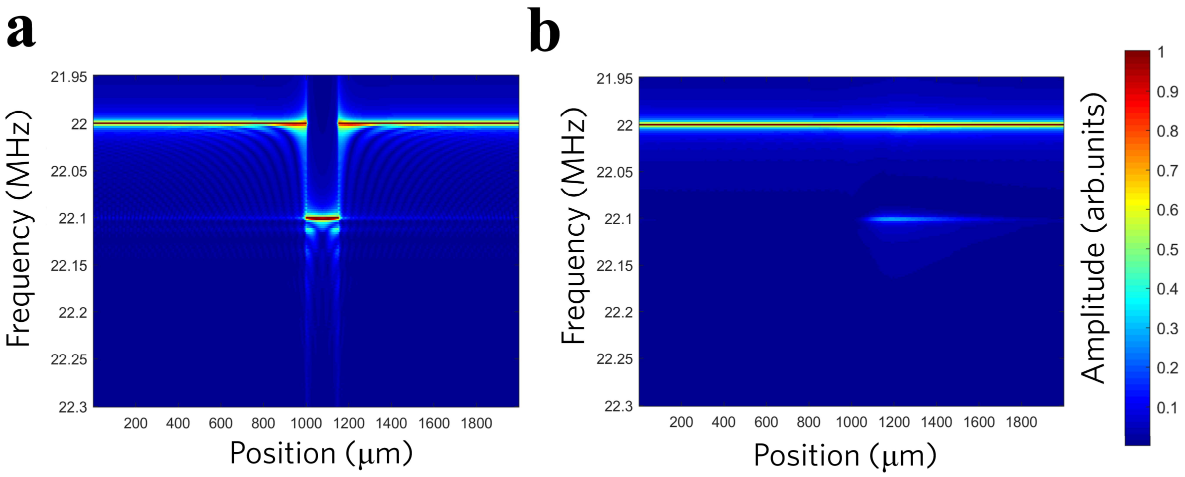

Supplementary Figure 1. Effect of the acoustic source size on the spatial resolution capability. The step of the resonant frequency is $\Delta\omega_{\mathrm{res}}$=100 kHz, and its width is 200 μm. The size of the optoacoustic source is 10 μm in (a) and 800 μm in (b).

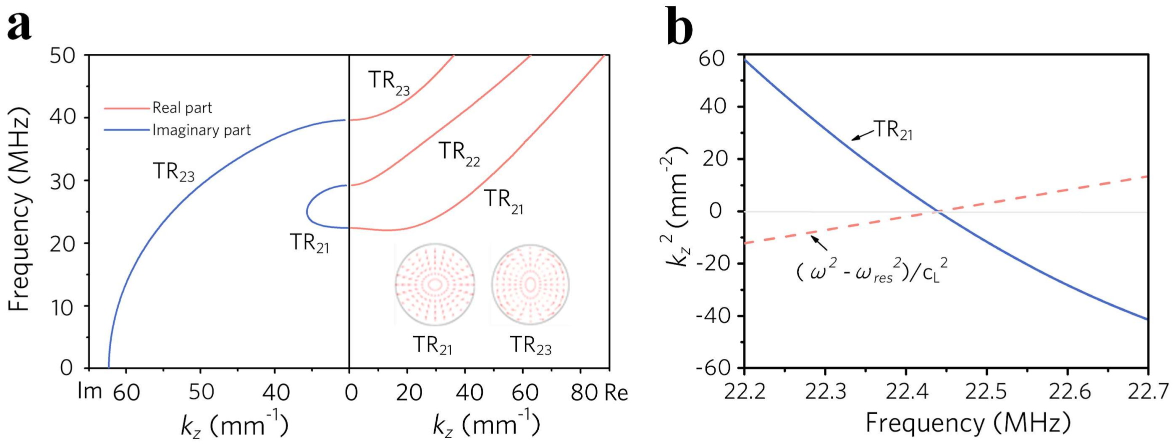

Supplementary Figure 2. Dispersion curves of the torsional-radial TR_{2n} modes. (a) Dispersion diagrams of the lowest-order torsional-radial (TR_{2n}) modes. Inset: mode profiles of the TR_{21} and TR_{23} modes. The arrows represent the amplitudes and directions of displacements. The interplay of the TR_{21} and TR_{22} modes forms an enclosed dispersion curve in the evanescent wave regime. It also significantly changes the dispersive property of the TR_{21} mode. (b) Plot of k_z^2 as a function of acoustic frequency. In contrast to the non-dispersive assumption (red dashed curve), the TR_{21} mode has a slope of -7700 rad^2 m^{-2} s^{-1}, which induces a 12-fold narrower mode width.

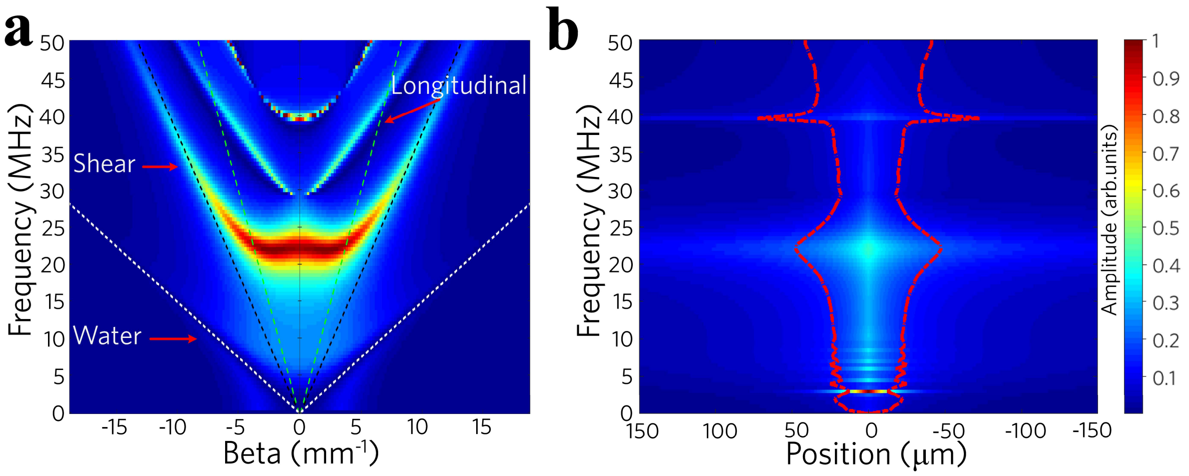

Supplementary Figure 3. Calculated dispersion diagram (a) and axial mode profile versus acoustic frequency (b) of the TR_{2n} modes in water.

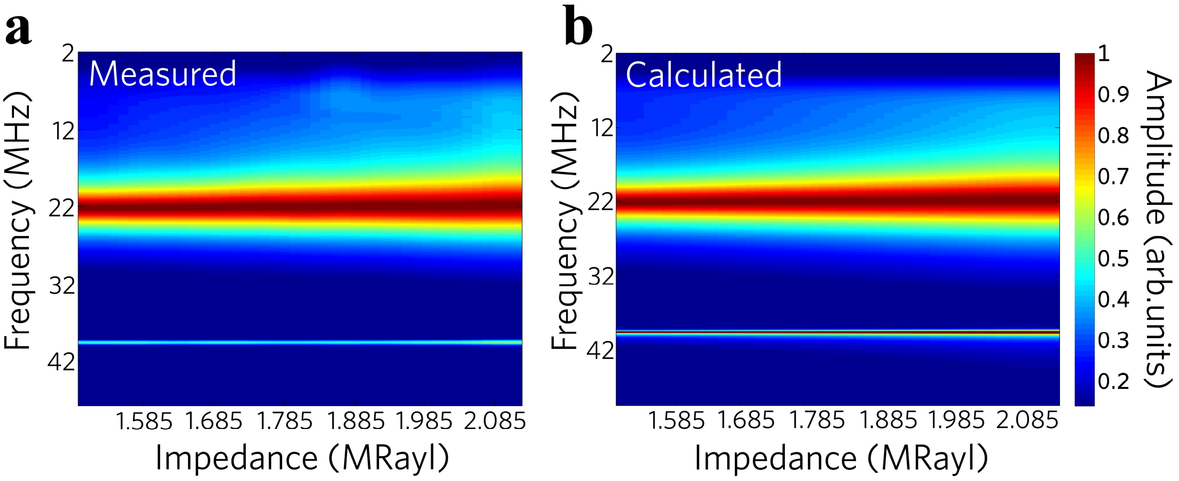

Supplementary Figure 4. Acoustic spectra as a function of surrounding impedance. (a) Measured result; (b) calculated result.

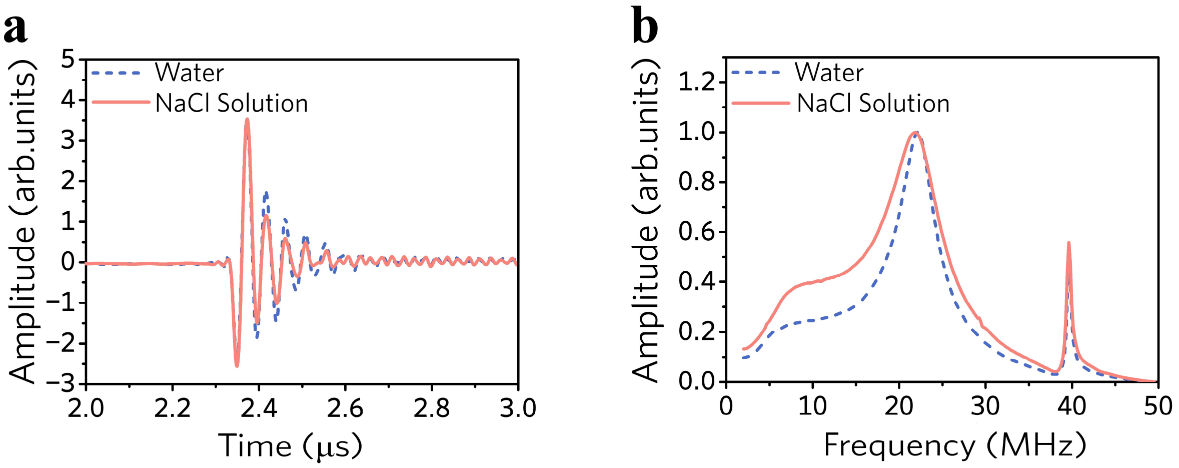

Supplementary Figure 5. Temporal waveforms and frequency spectra of the excited PTAVs in water and saturated NaCl solution. (a) Temporal waveforms and (b) frequency spectra of the excited acoustic vibration in water (acoustic impedance: Z=1.485 MRayl) and saturated NaCl solution (concentration: C=5.43 mol L^{-1}, acoustic impedance: Z=2.155 MRayl).

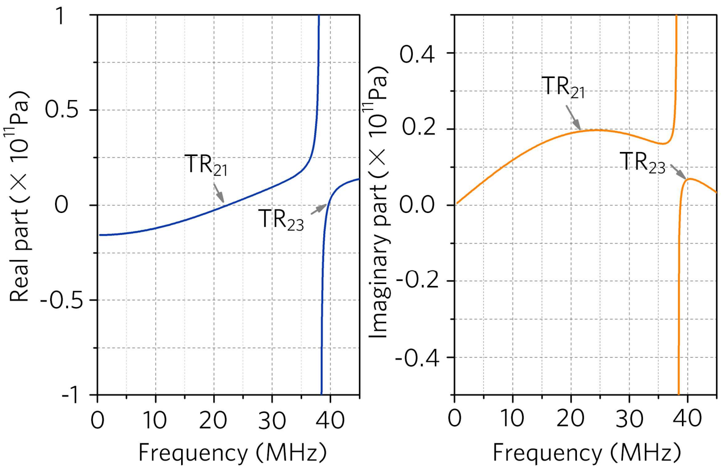

Supplementary Figure 6. Calculated real (left) and imaginary (right) parts in the denominator of Eq. S-21, analogous to frequency detuning and damping terms, respectively.

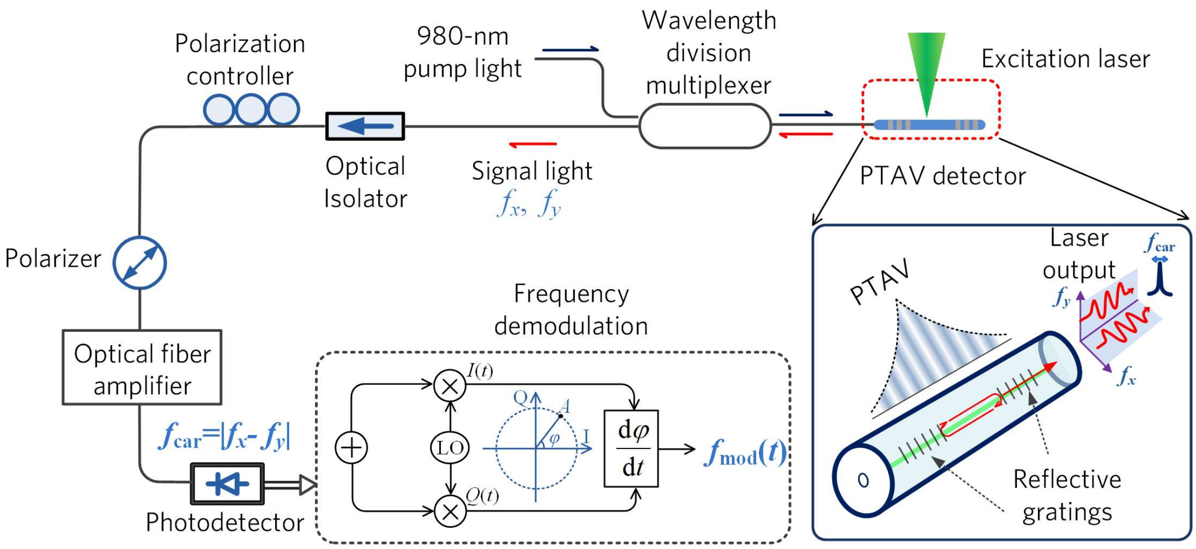

Supplementary Figure 7. Experimental setup for PTAV detection using the built-in laser.

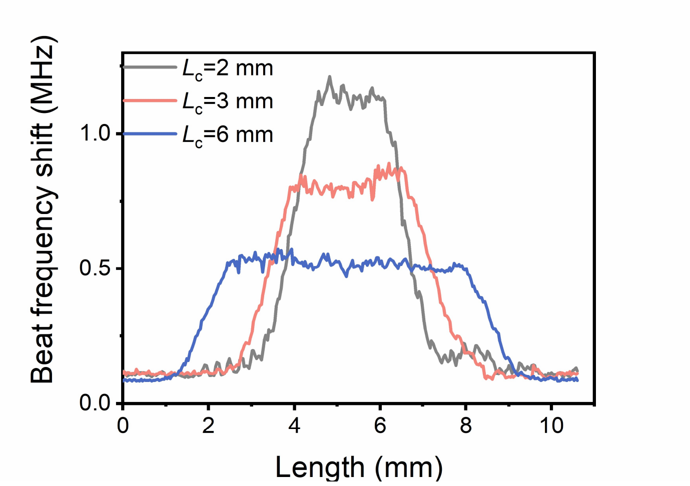

Supplementary Figure 8. Demonstration of the sensitivity/sensing length tradeoff of the PTAV detector.

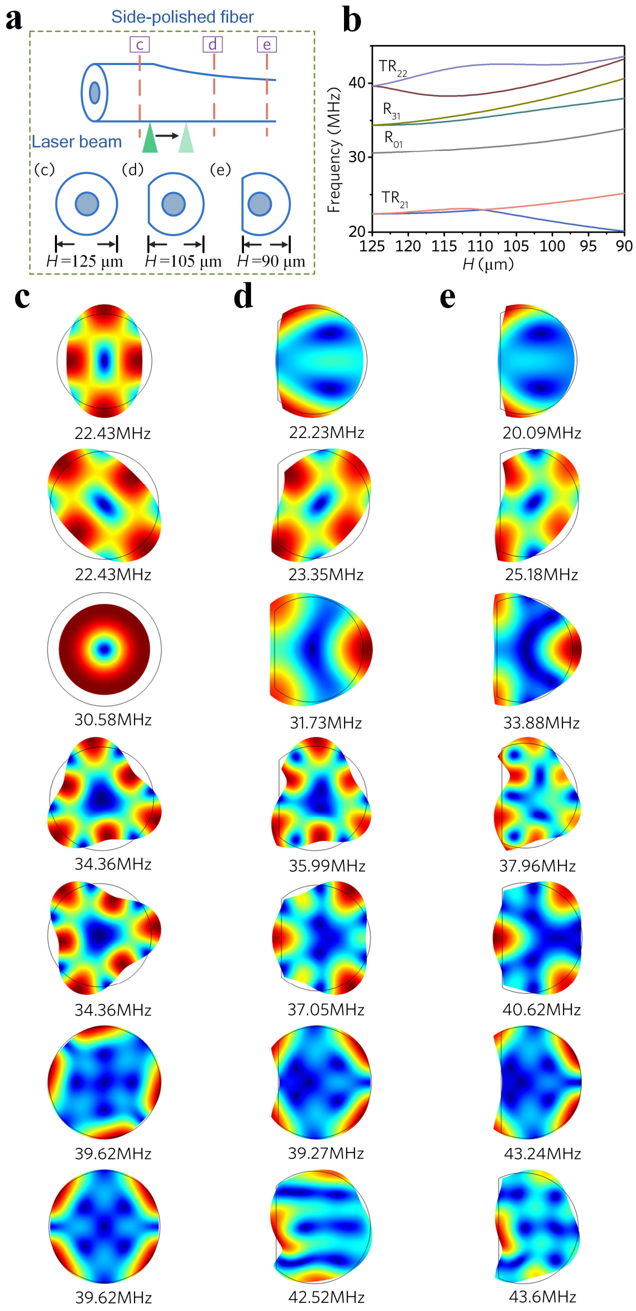

Supplementary Figure 9. Acoustic mode analysis of the side-polished fibre. (a) Schematic of the side-polished fibre, which contains a slow variation in the transverse geometry. (b) Calculated resonant frequencies as a function of *H*. (c-e) Acoustic mode profiles. Colour scale: amplitude of local displacement. The fibre has a slow variation in cross-sectional geometry, which is characterized by the diameter *H*. The TR_{21}, TR_{31} and TR_{23} modes are degenerated with a round geometry. The side polishing modifies the transverse geometry, changes the mode profiles and lifts the mode degeneracy.

Supplementary Table 1 Definition of terms for the calculation of fibre vibrational modes

φ	scalar acoustic potential
$\mathbf{H}$	vector acoustic potential
$\mathbf{u}$, u_r, u_θ	displacement and its radial/azimuthal components
σ_{rr}, $\sigma_{r\theta}$, σ_{rz}	stress components
Ω	acoustic angular frequency
c_w=1500 m s^{-1}	acoustic velocity in water
c_L=5878 m/s<br>c_S=3706 m/s	velocities of longitudinal/shear waves in a silica fibre
$k_{L,S}=\omega/c_{L,S}$	wavenumbers of longitudinal/shear waves in the fibre
ρ_w=1000 kg m^{-3}	water density
ρ_s=2240 kg m^{-3}	silica density
p_{44}=-0.0695	photoelastic coefficient

Supplementary Note 1 Optical-to-acoustic efficiencies of electrostriction and photothermal excitation.

Here, we perform a comparative study to estimate the optical-to-acoustic efficiencies of the electrostrictive force and photothermal excitation. The acoustic wave equation with a source can be generally expressed as

$$(\nabla^2 - \frac{1}{c_a}\frac{\partial^2}{\partial^2 t})p(\mathbf{r},t) = \mathbf{F}_{\mathrm{opt}} \quad \text{(S-1)}$$

where p denotes the acoustic wave and $\mathbf{F}_{\mathrm{opt}}$ is the optically induced acoustic source.

For electrostriction, the source is written as [1]

$$\mathbf{F}_{\mathrm{opt}} = \mathbf{F}_{\mathrm{es}} = -\frac{1}{2}\varepsilon_0\gamma_e\nabla^2|\mathbf{E}|^2 \quad \text{(S-2)}$$

where ε_0 represents the vacuum permittivity, γ_e=1.17 is the electrostrictive constant, and $\mathbf{E}$ is the electric field of the incident light. Substituting $|\mathbf{E}|^2 = \frac{2|\mathbf{S}|}{n_{\mathrm{si}}c\varepsilon_0}$, where $\mathbf{S}$ denotes the Poynting vector, n_{si}=1.45 is the refractive index of silica glass, and c is the velocity of light in vacuum, and considering that the spot size of the focused laser beam is approximately 10 μm, we can approximately obtain $\nabla^2 = k_{o\perp}^2 \cong \frac{1}{(1\times10^{-5})^2} = 10^{10}\ \mathrm{m}^{-2}$. Supposing that the intensity of the incident light is $|\mathbf{S}| = 1\ \mathrm{W\ m}^{-2}$, the electrostrictive force is estimated as $|\mathbf{F}_{\mathrm{es}}| = 30\ \mathrm{J\ m}^{-5}$.

For photothermal excitation, the acoustic source can be expressed as [2]

$$\mathbf{F}_{\mathrm{opt}} = \mathbf{F}_{\mathrm{pt}} = -\frac{\beta}{c_{\mathrm{p}}}\cdot\eta_{\mathrm{th}}\cdot\mu_{\mathrm{a}}\cdot\frac{\partial|\mathbf{S}|}{\partial t} \quad \text{(S-3)}$$

where $\eta_{\mathrm{th}} = 0.1$ represents the reflectivity of the gold coating, $\mu_{\mathrm{a}} = 6\times10^6\ \mathrm{m}^{-1}$ is the absorption coefficient, and β and c_p are the thermal expansion coefficient and thermal capacity, respectively. The first term can be written as $\frac{\beta}{c_{\mathrm{p}}} = \frac{\Gamma_{\mathrm{G}}}{v_{\mathrm{s}}}$, where $\Gamma_{\mathrm{G}} = 0.1$ is the Greunhauson parameter that characterizes the fraction of heat converted into mechanical vibration and v_{s} denotes the acoustic velocity in silica. Here, we take v_{s}=5878 m s^{-1} as the velocity of the longitudinal wave for simplicity. The last term in Equation S-3 can be approximately expressed as $\frac{\partial|\mathbf{S}|}{\partial t} = \omega_{\mathrm{bw}}|\mathbf{S}|$. Supposing $\omega_{\mathrm{bw}} = 2\pi\times20\ \mathrm{MHz}$ is the bandwidth corresponding to the pulse duration, the photothermally induced force with a 1 W m^{-2} intensity can be estimated as $|\mathbf{F}_{\mathrm{pt}}| = 2.13\times10^6\ \mathrm{J\ m}^{-5}$.

The above calculation suggests that the amplitude of the electrostrictive force is largely determined by the spatial gradient of the optical intensity. It depends on how tightly the light beam is focused. In contrast, photothermal excitation is highly efficient, taking advantage of the short pulse duration and the high absorptive coefficient of the gold coating. As a result, the photothermal effect can have an overwhelming optical-to-acoustic efficiency and induce a 10^5 to 10^6 times stronger force at the laser spot.

Supplementary Note 2 Modelling of the optoacoustic sensing: a matrix method.

Here, we develop a matrix method performed in the k_z domain for modelling of the optoacoustic sensing. A MATLAB code based on this method is provided. Calculations in the k_z domain can differentiate the mechanisms between F-SBS and the present PTAV-based sensing.

Suppose a nonuniform optical fibre to be measured is characterized by axial variation $\omega_{\mathrm{res}}(z)$; the wave equation in the $\omega\sim k_z$ space domain can be expressed as

$$[c_{\mathrm{a}}^2 k_z^2 \delta\left(k_z - k_z^{'}\right) + W(k_z) *] \cdot \Phi(\omega, k_z^{'}) - [\omega^2 + 2i\omega b] \cdot \Phi(\omega, k_z^{'}) = F(\omega, k_z) \quad \text{(S-4)}$$

where $W(k_z)$ and $F(\omega, k_z)$ are the k_z-domain Fourier transformation of axial variation $\omega_{\mathrm{res}}^2(z)$ and the external source in $\omega\sim k_z$ space, $\Phi(\omega, k_z^{'})$ denotes the solution in the $k_z^{'}$ domain, and "$*$" represents the convolution operator. The first step is to convert Equation S-4 into matrix form by quantizing the k_z and ω domains into N segments. A larger quantization number enables better frequency and spatial resolutions, but the calculation is more time consuming. Thus, $W(k_z)$, $F(\omega, k_z)$ and $\Phi(\omega, k_z^{'})$ at a selected frequency ω can all be written as N-element vectors. Now, the wave equation can be represented in matrix form:

$$\mathbf{S\Phi} = \mathbf{F} \quad \text{(S-5)}$$

where $\mathbf{S} = \mathbf{E}_{\mathrm{dig}} + \mathbf{W} - (\omega^2 + 2i\omega b) \cdot \mathbf{I_N}$, where $\mathbf{E}_{\mathrm{dig}}$ is a diagonal matrix with the ith element equal to $c_{\mathrm{a}}^2 k_{z,i}^2$. The term "$W(k_z) *$" is expressed as a Toeplitz matrix $\mathbf{W}$ with shifting $W(k_z)$ profile among each column, and its diagonal elements are all $V(0)$. Given a known vector $\mathbf{F}$ that describes the acoustic source, the solution can be calculated via $\mathbf{\Phi} = \mathbf{S^{-1}F}$.

Now, we consider the excitation and detection of the acoustic vibration to model PTAV-based optoacoustic sensing. The acoustic vibration in an optical fibre is detected via integral measurement of the induced optical phase change. The phase integral is equivalent to taking the $k_z^{'}$=0 component of $\Phi\left(\omega, k_z^{'}\right)$, assuming an infinitely long fibre. As a result, the frequency spectrum can be calculated with $\Phi\left(\omega, k_z^{'} = 0\right) = S^{-1}\left(\omega, k_z, k_z^{'} = 0\right) F(\omega, k_z)$ by scanning the frequency ω. Furthermore, the scanning point source can be expressed as $F(\omega, k_z, \Delta z) = \exp\left(-ik_z \cdot \Delta z\right)$, where $\Delta z = z^{'} - z$ denotes the scanning position with respect to the laser spot (acoustic source); then, we have

$$\mathbf{\Phi} = \mathbf{S^{-1}} \begin{pmatrix} \cdots \\ \exp\left(-ik_{z,-1} \cdot \Delta z\right) \\ \exp\left(-ik_{z,0} \cdot \Delta z\right) \\ \exp\left(-ik_{z,1} \cdot \Delta z\right) \\ \cdots \end{pmatrix} \quad \text{(S-6)}$$

Equation S-6 suggests that the solution is equivalent to an inverse Fourier transform of $\mathbf{S^{-1}}$ back to the z domain. Finally, we can obtain the result of $\Phi(\omega, \Delta z)$ by scanning the position Δz.

We would like to discuss the physics behind the matrix form in Equation S-5. For a nonuniform fibre, a series of eigenmodes is defined by $\left(\mathbf{E}_{\mathrm{dig}} + \mathbf{W}\right)\mathbf{X} = \omega^2\mathbf{X}$. The individual eigenvectors are $\mathbf{X_1}, \mathbf{X_2} \cdots \mathbf{X}_N$, and the eigenvalues are $\omega_1^2, \omega_2^2, \cdots \omega_N^2$. Because $\left(\mathbf{E}_{\mathrm{dig}} + \mathbf{W}\right)$ is a symmetric matrix, the left side of Equation S-5 can be extended as $\mathbf{S\Phi} = \mathbf{Q\Lambda Q^T} + (\omega^2 + 2i\omega b)\mathbf{I}_N$, where $\mathbf{Q} = (\mathbf{X_1}, \mathbf{X_2} \cdots \mathbf{X}_N)$ is composed of the individual eigenvectors, $\mathbf{Q^T} = \begin{pmatrix} \mathbf{X_1^T} \\ \mathbf{X_2^T} \\ \cdots \\ \mathbf{X_N^T} \end{pmatrix}$ is the transpose (and inverse matrix) of $\mathbf{Q}$, and $\mathbf{\Lambda}$ is a diagonal matrix composed of all the eigenvalues. The product $\mathbf{S\Phi}$ can be further written as $\mathbf{S\Phi} = \mathbf{Q\Lambda_c Q^T \Phi}$, where $\mathbf{\Lambda_c} = \mathbf{\Lambda} + (\omega^2 + 2i\omega b)\mathbf{I}_N$, considering $\mathbf{I}_N = \mathbf{QQ^T}$. The solution can be extended as a linear summation of each eigenmode as $\mathbf{\Phi} = \sum_N u_i \mathbf{X}_i$, and the product $\mathbf{Q^T\Phi}$ yields a vector $\mathbf{U} = \begin{pmatrix} u_1 \\ u_2 \\ \cdots \\ u_N \end{pmatrix}$. Based on Equation S-5, this vector $\mathbf{U} = \mathbf{\Lambda_c^{-1}}(\mathbf{Q^T F})$, with each entry $u_i = \mathbf{\Phi}_i^{\mathbf{T}}\mathbf{F} \cdot \frac{1}{\omega_i^2 - \omega^2 - 2i\omega b}$, is the frequency spectrum, with $\mathbf{\Phi}_i^{\mathbf{T}}\mathbf{F}$ the excitation efficiency of this mode. Therefore, we know that the matrix form in Equation S-5 corresponds to the calculation of the PTAV as a linear summation of the individual eigenmodes with a given acoustic source in the k_z domain.

Here, we would like to differentiate F-SBS and PTAV-based optoacoustic sensing based on the matrix method. We set a Gaussian-shaped acoustic source in the MATLAB code. Here, $\mathbf{F}$ is the Fourier transformation of the axial profile of the source. Supplementary Figure 1 suggests that a photothermally induced acoustic source

(10 μm in size) can well reconstruct the target $\omega_{\mathrm{res}}(z)$ with a step change of 100 kHz and a width of 200 μm. In contrast, when the size of the acoustic source is increased to 800 μm, the step change can hardly be reconstructed. For F-SBS, fibres tens of metres long are typically needed due to the weak electrostrictive excitation. As a result, the high-k_z components of the acoustic modes cannot be effectively excited for accurate reconstruction of the microstructure in the fibre.

Supplementary Note 3 Vibrational modes of a cylindrical optical fibre.

The acoustic vibration in an elastic cylinder can be described by the scalar and vectorial potentials φ and **H**, which satisfy the wave equations [3] (See the definition of terms in Supplementary Table 1)

$$\frac{1}{c_{\mathrm{L}}^2}\frac{\partial^2\varphi}{\partial^2 t} = \nabla^2\varphi \tag{S-7a}$$

$$\frac{1}{c_{\mathrm{S}}^2}\frac{\partial^2\mathbf{H}}{\partial^2 t} = \nabla^2\mathbf{H} \tag{S-7b}$$

The wave equation solutions are

$$\begin{cases} \varphi = A_l J_l(k_{r\mathrm{L}}r)\cos(l\theta)\exp(-ik_z z) \\ H_z = B_l J_l(k_{r\mathrm{S}}r)\sin(l\theta)\exp(-ik_z z) \\ H_\theta = D_l J_{l+1}(k_{r\mathrm{S}}r)\cos(l\theta)\exp(-ik_z z) \\ H_r = -D_l J_{l+1}(k_{r\mathrm{S}}r)\sin(l\theta)\exp(-ik_z z) \end{cases} \tag{S-8}$$

where J_l is the l-th order first-kind Bessel function, A_l, B_l and D_l are complex amplitudes, l=0, 1, 2 …. denotes the azimuthal mode order, and $k_{r\mathrm{L}} = \sqrt{k_{\mathrm{L}}^2 - k_z^2}$ and $k_{r\mathrm{S}} = \sqrt{k_{\mathrm{S}}^2 - k_z^2}$ are the radial wavenumbers. The time-dependent factor exp(iωt) is common to all expressions representing waves and is dropped for simplification. The relation between the displacement and the potentials is

$$\mathbf{u} = \nabla\varphi + \nabla\times\mathbf{H} \tag{S-9}$$

In cylindrical coordinates, the stress-displacement relations are given by

$$\sigma_{rr} = \lambda\nabla\cdot\mathbf{u} + 2\mu\frac{\partial u_r}{\partial r} \tag{S-10a}$$

$$\sigma_{r\theta} = \mu\left(\frac{1}{r}\frac{\partial u_r}{\partial\theta} + r\frac{\partial}{\partial r}\frac{u_\theta}{r}\right) \tag{S-10b}$$

$$\sigma_{rz} = \mu\left(\frac{\partial u_r}{\partial z} + \frac{\partial u_z}{\partial r}\right) \tag{S-10c}$$

For free vibration, σ_{rr}, $\sigma_{r\theta}$ and σ_{rz} should all be zero at the boundary, which gives

$$\mathcal{M}_{3\times3}\begin{pmatrix} A_l \\ B_l \\ D_l \end{pmatrix} = 0 \tag{S-11}$$

where $\mathcal{M}_{3\times3} = \begin{pmatrix} a_{11} & a_{12} & a_{14} \\ a_{21} & a_{22} & a_{24} \\ a_{41} & a_{42} & a_{44} \end{pmatrix}$ is the coefficient matrix, whose elements are $a_{11} = \gamma(\Omega_{\mathrm{L}}^2 + k_z^2 r^2)J_l(\Omega_{\mathrm{L}}) - 2J_l''(\Omega_{\mathrm{L}})$, $a_{12} = 2l\left[J_l'(\Omega_{\mathrm{S}}) - J_l(\Omega_{\mathrm{S}})\right]$, $a_{14} = 2ik_z rJ_{l+1}'(\Omega_{\mathrm{S}})$, $a_{21} = 2l\left[J_l'(\Omega_{\mathrm{L}}) - J_l(\Omega_{\mathrm{L}})\right]$, $a_{22} = -J_l''(\Omega_{\mathrm{S}}) + J_l'(\Omega_{\mathrm{S}}) - l^2 J_l(\Omega_{\mathrm{S}})$, $a_{24} = ik_z r\left[J_{l+1}'(\Omega_{\mathrm{S}}) - (l+1)J_{l+1}(\Omega_{\mathrm{S}})\right]$, $a_{41} = 2ik_z rJ_l'(\Omega_{\mathrm{L}})$, $a_{42} = -ik_z rlJ_l(\Omega_{\mathrm{S}})$, and $a_{44} = k_z^2 r^2 J_{l+1}(\Omega_{\mathrm{S}}) + J_{l+1}''(\Omega_{\mathrm{S}}) + (l+1)J_{l+1}'(\Omega_{\mathrm{S}}) - (l+1)J_{l+1}(\Omega_{\mathrm{S}})$, where $\Omega_{\mathrm{L}}=k_{\mathrm{L}}a$, $\Omega_{\mathrm{S}}=k_{\mathrm{S}}a$, and $\gamma=\lambda/\mu$, with λ and μ the Lamé elastic constants for compressibility and shear modulus. The equations have nonzero solutions when the determinant is $|\mathcal{M}_{3\times3}| = 0$, yielding a discrete set of dispersion curves. Supplementary Figure 2a plots the dispersion curves of the lowest-order $\mathrm{TR}_{2\mathrm{n}}$ modes over both the real (propagation wave) and imaginary (evanescent wave) regimes. For the calculation of the transverse mode with k_z=0, the coefficient matrix degenerates to $\mathcal{M}_{2\times2} = \begin{pmatrix} a_{11} & a_{12} \\ a_{21} & a_{22} \end{pmatrix}$. With a determinant of $|\mathcal{M}_{2\times2}| = 0$, the eigenmodes defined by the transverse geometry can all be determined. The displacement profiles of the TR_{21} and TR_{23} modes are illustrated in Supplementary Figure 2. For the l=0 modes, i.e., the axially symmetric modes, B_l=0, and only the compressional waves exist. The matrix further degenerates to $\mathcal{M} = a_{11}$, which has been used for mode calculations in refs. [4, 5].

Supplementary Figure 2 suggests that the individual dispersion curves have different shapes. For example, the TR_{23} mode has a profile that is close to the scalar wave case with $k_z = \sqrt{\frac{\omega^2}{c_{\mathrm{a}}^2} - k_{\perp}^2}$. The TR_{21} mode, in contrast, presents the minimum frequency at approximately k_z=15 mm^{-1}. Between k_z=0 and 15 mm^{-1}, the dispersion curve has a negative slope as a result of the interplay between the TR_{21} and TR_{22} modes. These two modes emerge in the evanescent wave regime, forming an enclosed dispersion curve. This unique dispersive property of the TR_{21} mode can significantly change the complex wavenumber γ as well as the longitudinal profiles of the PTAVs. To better understand the dispersion effect, we plot k_z^2 as a function of acoustic frequency in Supplementary Figure 2b. Compared with the non-dispersive curve $k_z^2 = \frac{\omega^2 - \omega_{\mathrm{res}}^2}{c_{\mathrm{L}}^2}$ with a slope of 51.5 rad^2 m^{-2} s^{-1}, the TR_{21} mode curve has a slope of -7700 rad^2 m^{-2} s^{-1}. The negative sign means that the frequencies higher than ω_{res} correspond to the evanescent wave and the lower frequencies correspond to propagation waves. The effective acoustic velocity of the TR_{21} mode at ω_{res} is only 480 m s^{-1}. Such a dispersion curve can effectively induce a 12-fold narrower PTAV spatial width, based on Equation 1.

Supplementary Note 4 Calculation of the sensitivity to a thickness change of the coating based on perturbation theory.

Here, a perturbation theory is developed to estimate the transverse vibration response in terms of a resonance shift with changing overlay thickness. We start with the wave equation expressed with displacement $\mathbf{u}$ [3],

$$\frac{\partial^2 \mathbf{u}}{\partial t^2} = c_\mathrm{S}^2 \nabla^2 \mathbf{u} + (c_\mathrm{L}^2 - c_\mathrm{S}^2)\nabla \cdot (\nabla \mathbf{u}) \tag{S-12}$$

Notably, both acoustic velocities are related to the material density, with $c_\mathrm{S}^2 = \frac{1}{\rho}\frac{E}{2(1+2\sigma)}$ and $c_\mathrm{L}^2 = \frac{1}{\rho}\frac{E(1-\sigma)}{(1+\sigma)(1-2\sigma)}$, where E and σ are the Young's modulus and Poisson ratio of silica glass. Multiplying both sides of Eq. S-6 by ρ and writing $\frac{\partial^2 \mathbf{u}}{\partial t^2}$ as $-\omega_\mathrm{res}^2 \mathbf{u}$, we have

$$-\rho\omega_\mathrm{res}^2 \mathbf{u} = \rho c_\mathrm{S}^2 \nabla^2 \mathbf{u} + \rho(c_\mathrm{L}^2 - c_\mathrm{S}^2)\nabla \cdot (\nabla \mathbf{u}) \tag{S-13}$$

The right side of Eq. S-13 is simply written as $\mathcal{L}\mathbf{u}$, which is a linear operator. By introducing a geometric perturbation $\delta\rho(r,\theta)$ to the existing transverse geometry (r,θ), the resonant frequency and the displacement field change to $\omega_\mathrm{res}^2 + \delta\omega_\mathrm{res}^2$ and $\mathbf{u} + \delta\mathbf{u}$, respectively, which satisfy

$$-(\rho + \delta\rho)(\omega_\mathrm{res}^2 + \delta\omega_\mathrm{res}^2)\,(\mathbf{u} + \delta\mathbf{u}) = \mathcal{L}(\mathbf{u} + \delta\mathbf{u}) \tag{S-14}$$

By subtracting Eq. S-13 and ignoring the second- and third-order perturbations, Eq. S-14 can be rewritten for the mth mode as

$$(\mathcal{L} + \rho\omega_\mathrm{res}^2)\delta\mathbf{u}_m = -(\rho\delta\omega_\mathrm{res}^2 + \delta\rho\omega_\mathrm{res}^2)\mathbf{u}_m \tag{S-15}$$

Expanding the modification to the displacement as $\mathrm{d}\mathbf{u}_m = \sum_n a_{mn}\,\mathbf{u}_n(r,\theta)$, the left side changes into a summation $\sum_n a_{mn}\,(\mathcal{L} + \rho\omega_\mathrm{res}^2)\mathbf{u}_n(r,\theta)$. Multiplying both sides by $\mathbf{u}_m^*(r,\theta)$ and integrating over the whole transverse geometry, the left side vanishes, leaving only the right side, which is further expressed as

$$\delta\omega_\mathrm{res} = -\frac{\omega_\mathrm{res}}{2}\frac{\iint \mathbf{u}_m^* \cdot \delta\rho(r,\theta) \cdot \mathbf{u}_m \mathrm{d}r\mathrm{d}\theta}{\iint \mathbf{u}_m^* \cdot \rho(r,\theta) \cdot \mathbf{u}_m \mathrm{d}r\mathrm{d}\theta} \tag{S-16}$$

Substituting the densities of silica glass and the coating material ρ_s and ρ_c, as well as the fibre radius r_0 and coating thickness d_c ($d_\mathrm{c} << r_0$), the coating-induced resonant frequency change can be written as

$$\delta\omega_\mathrm{res} = -\omega_\mathrm{res}\frac{\pi a d_\mathrm{c}\rho_\mathrm{c} \cdot \mathbf{u}_m^*|_{r=a} \cdot \mathbf{u}_m|_{r=a}}{\rho_s \int_0^a \int_0^{2\pi} \mathbf{u}_m^* \cdot \mathbf{u}_m \mathrm{d}r\mathrm{d}\theta} \tag{S-17}$$

With the radial and azimuthal displacements $\mathbf{u}_{21}(r,\theta)$ of the TR_{21} mode based on the theory in Supplementary Note 3, we can calculate that a gold coating (with a density of 1.93×10^4 kg m^{-3}) can shift the resonant frequency by 2 kHz nm^{-1}.

Supplementary Note 5 Optoacoustic sensing in liquids.

Now consider the PTAVs damped by a liquid medium. Supplementary Figure 3 shows the dispersion diagram as well as the spatial mode profile of the PTAVs in water. The TR_{21} mode curve significantly broadens as a result of the strong acoustic interaction with the surrounding medium. The spectral recovery though fibre-optic detection still applies, which has been verified by the calculated mode profiles. Therefore, the acoustic spectra can be calculated with a two-dimensional model, supposing an invariant acoustic structure along the axial direction. Note that the fibre vibration can exert pressure waves, which are depicted as outwards propagating cylindrical waves $C_l H_l^{(1)}(k_\mathrm{a} r)$, where $H_l^{(1)}$ represents the outwards propagating pressure wave and C_n denotes its amplitude. The interaction between the solid fibre and surrounding medium can be depicted by the following linear equations, S-12 (a-c), based on the boundary conditions. The continuity of the radial stress/pressure can be expressed as [4]

$$a_{11}A_l + a_{12}B_l + a_{13}C_l = b_1 \tag{S-18a}$$

where $a_{13} = H^{(1)}(Z)/\rho_\mathrm{S}\omega^2$ and b_1=0. Considering that shear waves are not supported in the fluidic medium, the expression presenting zero shear stress at the boundary still holds and can be rewritten as

$$a_{21}A_l + a_{22}B_l + a_{23}C_l = b_2 \tag{S-18b}$$

where a_{23}=0 and b_2=0. In addition, the continuity of radial displacement demands

$$a_{31}A_l + a_{32}B_l + a_{33}C_l = b_3 \tag{S-18c}$$

where $a_{31} = -J_l'(Z_\mathrm{L})$, $a_{32} = lJ_l\ (Z_\mathrm{S})$ and $a_{33} = -H_l^{(1)\prime}(Z)/\rho_\mathrm{S}\omega^2$. Assume that the radial displacement created by the acoustic dipole source can be simply written as $b_3\cos(l\theta)$. The amplitude of b_3 is related to the optical absorption, thickness of the absorptive layer, irradiation area and elastic properties of silica glass. However, the amplitudes do not affect the acoustic spectrum and will not be analysed in detail in this context. Combining Eqs. S-18 (a-c), the coefficients A_l, B_l, and C_l can be solved. The response in terms of the beat frequency variation Δf_b is proportional to the birefringence change described as $\Delta f_\mathrm{b} = \frac{c}{n_\mathrm{eff}\lambda} \cdot \Delta B$. The birefringence change ΔB is determined by

$$\Delta B = -p_{44}n_0^3 A_l \cdot \frac{k_\mathrm{L}^2 + \frac{a_{31}}{a_{33}}k_\mathrm{S}^2}{2} \tag{S-19}$$

Substituting $A_l = \frac{\det(M)}{\det(N)}$, where $M = \begin{pmatrix} b_1 & a_{12} & a_{13} \\ b_2 & a_{22} & a_{23} \\ b_3 & a_{32} & a_{33} \end{pmatrix}$ and $N = \begin{pmatrix} a_{11} & a_{12} & a_{13} \\ a_{21} & a_{22} & a_{23} \\ a_{31} & a_{32} & a_{33} \end{pmatrix}$, into Eq. S-19, the optical response can be expressed as

$$\Delta f_\mathrm{b} = -\frac{c}{n_\mathrm{eff}\lambda} \cdot \frac{p_{44}n_0^3}{2} \cdot \frac{\det(M)}{\Gamma \cdot \det(N)} \tag{S-20}$$

where $\Gamma = \frac{1}{k_\mathrm{L}^2 + \frac{a_{31}}{a_{33}}k_\mathrm{S}^2}$. Supplementary Figure 4 plots the calculated and measured spectrograms with varying surrounding impedance from 1.485 MRayl (deionized water) to 2.155 MRayl (NaCl solution), which are in good agreement with the experimental results. The impedance response was measured by exciting the PTAV with a stationary laser beam and gradually changing the concentration of the NaCl solution. The TR_{21} and TR_{23} modes present significantly different 3-dB bandwidths, 6.34 and 0.36 MHz, and broaden at different rates as a result of the strength difference in the acoustic interaction with the surrounding medium. Supplementary Figure 5a shows the recorded acoustic waveforms when submerged in deionized water (Z=1.485 MRayl) and saturated NaCl solution (Z=2.155 MRayl). Supplementary Figure 5b exhibits the corresponding frequency responses, which are normalized to the peak amplitude of the TR_{21} mode. The acoustic signal decays faster at higher impendence (decay rate from 9.7 to 15.5 μs^{-1}), resulting in broadened TR_{21} resonance as a result of the stronger dissipation to the surrounding medium. In contrast, the TR_{23} mode is hardly sensitive to the impedance due to the much weaker interaction with the surrounding medium. This is a result of the minimal radial displacement at the fibre surface, which originates from the cancellation in the radial direction (see Supplementary Figure 2).

The impedance response can be understood based on a simplified resonator model. The acoustic spectrum of the damped fibre is largely determined by the term $\Gamma \cdot \det(N)$ in the denominator, which is extended as $\Gamma \cdot \det(N) = \rho_\mathrm{S} c_\mathrm{L} \Gamma \begin{vmatrix} a_{11} & a_{12} \\ a_{21} & a_{22} \end{vmatrix} H_l'^{(1)}(Z) + \rho_\mathrm{w} c_\mathrm{w} \Gamma \begin{vmatrix} a_{21} & a_{12} \\ a_{31} & a_{32} \end{vmatrix} H_l^{(1)}(Z)$. Considering the phase relation between the Hankel function and its derivative (90 degrees in advance), these two terms are analogous to detuning and damping terms, respectively.

Supplementary Figure 6 shows the amplitudes of these two terms. At frequencies of 22.3 and 39.6 MHz with the detuning term approaching zero, the acoustic response reaches a maximum. The damping term contains $\rho_\mathrm{w} c_\mathrm{w}$, suggesting that the radiation loss is proportional to the acoustic impedance of the surrounding medium. The spectral width can be approximately expressed as

$$\Delta f_{\mathrm{FWHM}} = \frac{\rho_{\mathrm{W}} c_{\mathrm{W}}}{\rho_{\mathrm{S}} c_{\mathrm{L}}} \cdot \frac{\begin{vmatrix} a_{21} & a_{12} \\ a_{31} & a_{32} \end{vmatrix}}{\begin{vmatrix} a_{11} & a_{12} \\ a_{21} & a_{22} \end{vmatrix}} \tag{S-21}$$

The first term of Eq. S-21 suggests that the spectral width is proportional to the impedance contrast between the two media. The second term suggests its relation to the vibration mode property, which is determined by the parameters including acoustic velocities and material densities. Comparing the TR_{21} and TR_{23} modes, we found that the effective slopes of detuning are 4.88×10^{8} and 1.11×10^{10} Pa MHz^{-1} and the damping rates are 1.95×10^{9} and 6.23×10^{8} Pa, respectively. As a result, the spectral width of the TR_{23} peak is approximately 70-fold narrower than that of the TR_{21} peak. This suggests a much weaker interaction between the TR_{23} mode and the surrounding medium. Both the detuning and damping terms approach infinity at 38.4 MHz as a result of the infinitely large Γ. This corresponds to the Fano resonance between the TR_{21} and TR_{23} modes.

Supplementary Note 6 PTAV detection by using the built-in fibre laser.

Supplementary Figure 7 shows the detailed experimental setup for PTAV detection. The detector is fabricated in an Er/Yb codoped fibre by photoinscribing two highly reflective distributed index gratings. It lases at 1530 nm at both x- and y-polarizations when pumped with a 980-nm laser diode via a wavelength-division multiplexer. The output power is approximately 500 μW and is amplified by using an erbium-doped fibre amplifier (EDFA) to 28 mW when launching at the photodetector (Discovery Semiconductor, DSC50S) to maximize the signal-to-noise ratio. We mixed the frequency-modulated signal with a local high-purity microwave signal for frequency down-conversion to a radiofrequency of approximately 100 MHz. An I/Q demodulation system measures its phase change and reconstructs the frequency modulation by conducting a first-order time derivation. The sampling rate is 100 MHz in the experiment, which gives an acquisition bandwidth of 50 MHz.

The fibre typically has a weak intrinsic birefringence, which creates a frequency difference between the two orthogonal lasing modes (f_x and f_y) and creates a beating signal at f_{car}=2.2 GHz (carrier signal). The photothermally induced vibration induces a frequency modulation of the output beat signal between the two orthogonal polarized lasing modes [6]. A polarization controller and an in-line polarizer are used to maximize the beat signal. The beat frequency of the PTAV detector is $f_b=cB/n_0\lambda$, where c denotes the speed of light in vacuum, n_0 is the refractive index of silica glass, λ is the lasing wavelength, and B is the fibre birefringence. The frequency modulation induced by the acoustic variation writes [6, 7]

$$f_{mod}=\frac{c}{n_0\lambda}\cdot\Delta B\cdot\frac{L_s}{L_c} \qquad \text{(S-22)}$$

where ΔB represents the local birefringence change at the laser spot, L_c represents the cavity length, and L_s denotes the effective PTAV width, defined by $L_s=\int_{-\infty}^{\infty}\kappa u(z)\mathrm{d}z/\Delta B$, where $u(z)$ represents the spatial profile of the acoustic vibration and κ the effective elastic-optic coefficient for optical birefringence. The acoustic source should reside along the principle axis of the fibre to maximize the induced birefringence change and the resultant optical response.

Supplementary Figure 8 demonstrate the measured beat frequency changes induced by the PTAVs with different cavity lengths L_c=2, 3, and 6 mm, respectively. The measurement was performed by scanning the excitation laser while recording the waveforms of the acoustic vibrations along each detector in air. Here, the single pulse energy of the 532-nm laser is kept at 230 nJ. Based on Equation S-22, the sensitivity is inversely proportional to the cavity length L_c. The detector has a tradeoff between the sensing length and sensitivity.

Supplementary References

1. Boyd, R. W., Nonlinear Optics, Third Edition, 2008, Chapter 9, Elsevier (Singapore) Pte Ltd.
2. Wang, L. V. and Wu, H.-i, Biomedical Optics: Principles and Imaging, Chapter 12, Wiley.
3. Meeker, T. R., and Meitzler, A. H., Guided Waves Propagation in Elongated Cylinders and Plates. Physical Acoustics, W. P. Mason, ed., vol. I-Part A, pp. 111–167.
4. Flax L., Varadan, V. K., & Varadan, V. V. Scattering of an obliquely incident acoustic wave by an infinite cylinder. J. Acoust. Soc. Am. 68, 1832-1835 (1980).
5. Flax, L., Cole, J. H., De Paula, R. P., and Bucaro, J. A. Acoustically induced birefringence in optical fibres. *J. Opt. Soc. Am.* 72, 1159-1162 (1982).
6. Liang, Y., Jin, L., Wang, L., Bai, X., Cheng, L., and Guan, B.-O., Fibre-laser-based ultrasound sensor for photoacoustic imaging. *Scientific Reports* 7, 40849 (2017).
7. Zhou, F., Jin, L., Liang, Y., Cheng, L., and Guan, B.-O. Spatial sensitivity characterization of dual-polarization fibre grating laser sensors. *J. Lightwave Technol.* 33, 4151-4155 (2015).
