# Supplementary material for: High spatiotemporal resolution optoacoustic sensing with photothermally induced acoustic vibrations in optical fibres
Source: Nat Commun. 2021 Jul 6;12:4139. doi: 10.1038/s41467-021-24398-w (PMC8260642; doi:10.1038/s41467-021-24398-w)
Supplement: Supplementary file 3 — Description of Additional Supplementary Files [file 41467_2021_24398_MOESM3_ESM.docx]

**Description of Additional Supplementary Files**

File Name: Supplementary Movie 1

Description: Optoacoustic sensing result of the diffusion dynamics in the microfluidic channel. The PTAV detector is suspended in the common path (2 mm in width and 1 mm in height) of a Y-shaped diffusion cell (Figure 4a) for optoacoustic sensing. This movie shows the dynamic process of the impedance gradient between water and NaCl solution establishes (at t=3 s) and vanishes (at t=32 s). Upper left: acoustic spectrogram. Lower left: Measured acoustic impedance Z across the channel. Right: Impedance map over time. Frame rate: 50 Hz.
